# Effect of the Cut-Off Level for Thyroid-Stimulating Hormone on the Prevalence of Subclinical Hypothyroidism among Infertile Mexican Women

**DOI:** 10.3390/diagnostics11030417

**Published:** 2021-03-01

**Authors:** Lidia Arce-Sánchez, Salvatore Giovanni Vitale, Claudia Montserrat Flores-Robles, Myrna Souraye Godines-Enriquez, Marco Noventa, Carmen Marcela Urquia-Figueroa, Nayeli Martínez-Cruz, Guadalupe Estrada-Gutierrez, Salvador Espino y Sosa, José Romo-Yañez, Araceli Montoya-Estrada, Enrique Reyes-Muñoz

**Affiliations:** 1 Department of Endocrinology, Instituto Nacional de Perinatología “Isidro Espinosa de los Reyes”, Montes Urales 800, Mexico City 11000, Mexico; li_arce@yahoo.com.mx (L.A.-S.); cmontsefr@gmail.com (C.M.F.-R.); nayemc_21@hotmail.com (N.M.-C.); 2 Unit of Gynecology and Obstetrics, Department of General Surgery and Medical Surgical Specialties, Uni versity of Catania, 95123 Catania, Italy; sgvitale@unict.it; 3 Deputy Director of Education in Health Sciences, National Institute of Perinatology, Ministry of Health, Mexico City 11000, Mexico; dra.myrnagodines@gmail.com (M.S.G.-E.); marcela2709@hotmail.com (C.M.U.-F.); 4 Unit of Gynecology and Obstetrics, Department of Women and Children’s Health, University of Padua, 35122 Padua, Italy; marco.noventa.1@unipd.it; 5 Research Direction, Instituto Nacional de Perinatología, “Isidro Espinosa de los Reyes”, Montes Urales 800, Mexico City 11000, Mexico; gpestrad@gmail.com; 6 Clinical Research Branch, Instituto Nacional de Perinatología “Isidro Espinosa de los Reyes”, Montes Urales 800, Mexico City 11000, Mexico; salvadorespino@gmail.com; 7 Coordination of Gynecological and Perinatal Endocrinology, Instituto Nacional de Perinatología “Isidro Espinosa de los Reyes”, Montes Urales 800, Mexico City 11000, Mexico; jryz@yahoo.com (J.R.-Y.); ara_mones@hotmail.com (A.M.-E.)

**Keywords:** TSH, subclinical hypothyroidism, infertility, obesity

## Abstract

The primary aim of this study was to compare the prevalence of subclinical hypothyroidism (SCH) using two different cut-off levels for TSH values (≥2.5 mIU/L versus ≥4.1 mIU/L). The secondary objective was to analyze the clinical-biochemical characteristics in women with and without SCH. This was a retrospective cross-sectional study. In total, 1496 Mexican women with infertility were included: Group 1, women with TSH levels ranging between 0.3 and 2.49 mIU/L, *n* = 886; Group 2, women with TSH between 2.5 and 4.09 mIU/L, *n* = 390; and Group 3, women with TSH ≥4.1 mIU/L *n* = 220. SCH prevalence was 40.7% (CI 95%: 38.3–43.3%) with TSH cut-off ≥ 2.5 mIU/L, and 14.7% (CI 95%: 12.7–16.5%) with TSH cut-off ≥ 4.1 mIU/L, (*p* = 0.0001). The prevalence of overweight was higher in Group 2 than in Groups 1 and 3. Thyroid autoimmunity, obesity and insulin resistance were higher in Group 3 than in Group 1 (*p* < 0.05). No other differences were observed between groups. Conclusions: The prevalence of SCH in our selected patients increased almost three times using a TSH cut-off ≥ 2.5 mIU/L compared with a TSH cut-off ≥ 4.1 mIU/L. Women with TSH ≥4.1 mIU/L compared with TSH cut-off ≤ 2.5 mIU/L more often presented with obesity, thyroid autoimmunity and insulin resistance.

## 1. Introduction

Subclinical hypothyroidism (SCH), a mild form of hypothyroidism defined as elevated thyroid-stimulating hormone (TSH) with normal free thyroxine (fT4) levels [1], is a common diagnosis among women of reproductive age [2,3,4]. However, there is controversy around the definition of SCH and to whom treatment should be offered. This is observed particularly in women attempting to get pregnant [2,3].

In 2011, the American Thyroid Association published for the first time guidelines on the diagnosis and management of thyroid disease during pregnancy and recommended trimester-specific upper reference limits for TSH (first trimester, 2.5 mIU/L; second and third trimester 3.0 mIU/L) [4]. Many studies [1,5] published after 2011 showed that using these upper limits for defining SCH resulted in overdiagnosis of this condition, so in 2017, the American Thyroid Association (ATA) changed its recommendations, encouraged using TSH reference ranges obtained for the pregnant local population and proposed that when these are not available, 4 mIU/L be used as a TSH upper reference limit in early pregnancy [1].

In pregnancy, SCH and thyroid autoimmunity are associated with first-trimester miscarriage and adverse obstetric and neurodevelopmental outcomes; however, there is no evidence that these complications increase in women with TSH concentrations between 2.5 and 4 mIU/L without autoimmunity [1,2,3,4,5,6,7,8]. Currently, there is no consensus about whether to treat women with TSH concentrations at the high end of the normal range [1,2,3] because of both the controversial association with adverse perinatal outcomes and the lack of homogeneity and adequate control groups in available clinical trials [2,3,4,5,6,7,8,9,10,11,12,13,14,15,16].

The routine universal pre-conception thyroid testing is not recommended currently by any of the most important professional organizations such as the American Thyroid Association (ATA) [1], The American College of Obstetricians and Gynecologists (ACOG) [9], The National Institute for Health and Care Excellence (NICE) [10] and The American Society for Reproductive Medicine (ASRM) [3], primarily because large-scale randomized trials have not proven the benefits of this practice [7].

The prevalence of SCH in the general population ranges between 6 and 8% [9]. Given the lack of consensus on the selection of a TSH cut-off level for SCH diagnosis in the preconception period [1,3,8,12,13,14,15,16,17,18,19,20], the reported prevalence of this condition among infertile women is highly variable, ranging from 0.7 to 43% [21,22,23].

Some authors have suggested an association between SCH and infertility or reduced ovarian reserve [15,16], but their data are controversial due to the differences in the TSH cut-off proposed. The 2017 ATA Guidelines recommend TSH screening for all women seeking care for infertility [1], and despite insufficient existing evidence determining if levothyroxine (LT4) improves fertility in women with SCH, they suggest that low-dose LT4 (25–50 ug/d) be considered in these women, with TSH > 4 mIU/L, to prevent progression to overt hypothyroidism [1]. In the case of women undergoing assisted reproduction technologies (ART), one meta-analysis [22] reported that LT4 treatment improves clinical pregnancy outcomes (delivery rate) in women with SCH and/or thyroid autoimmunity. All clinical trials included in this meta-analysis defined SCH with a TSH higher than 4 mIU/L, so these conclusions could not be applied to infertile women with TSH levels between 2.5 and 4 mIU/L.

To elucidate the effect of diagnostic criteria of SCH in the infertile Mexican population, we designed this study with the primary aim of comparing the prevalence of SCH using two different cut-off levels for TSH values ≥ 2.5 mIU/L versus ≥4.1 mIU/L; as a secondary aim, we analyzed the clinical-biochemical characteristics among infertile Mexican women with and without SCH, using different TSH cut-off levels.

## 2. Materials and Methods

### 2.1. Study Design and Patients

We conducted a retrospective cross-sectional study on patients seeking fertility treatment at the Infertility Clinic of the National Institute of Perinatology Isidro Espinosa de los Reyes, in Mexico City. All women attending the Institute from 2010 to 2014 were enrolled. Patients underwent thyroid and hormonal profile determinations as part of the fertility testing. Infertility was defined as a couple reporting at least one year of active sex life without contraception aiming to achieve pregnancy. We excluded women with diabetes mellitus type 1, women with preexistent primary or secondary overt hypothyroidism (TSH > 10 mIU/mL and low fT4 concentrations) and/or hyperthyroidism, hyperprolactinemia, or incomplete clinical records. The data collection was obtained from clinical records, and biochemical data were obtained from the database of the Endocrinology Department. The Ethics and Research Committees of the National Institute of Perinatology Isidro Espinosa de los Reyes approved this study on 6 July 2015, with registry number 212250-2102-10209-01-15.

### 2.2. Data Collection

All women were evaluated for endocrine–ovarian function. The following variables were systematically recorded: hormonal profile on days 3–5 of the menstrual cycle, including TSH; total triiodothyronine (TT3); fT4; LH; FSH; estradiol (E2); and prolactin (PRL). Progesterone (P) was determined on days 21–23 of the natural or induced menstrual cycle. In women with suspected polycystic ovary syndrome (PCOS), the following androgen levels were measured: total testosterone (TT), sex hormone binding globulin (SHBG), androstenedione (Δ4), dehydroepiandrosterone sulfate (DHEA-S), 17-hydroxyprogesterone (17-OHP4). We also tested patients for the presence of anti-Thyroperoxidase antibodies (TPO-Abs) and anti-thyroglobulin antibody (TG-Abs). During the study period, thyroid antibodies were measured mainly in women with TSH ≥ 4.1 mU/L, and women with TSH ≤ 4 mIU/L were tested for antibodies only in the last two years, predominantly in women with TSH > 2.5 mIU/L. Finally, we measured fasting insulin and glucose levels and calculated the HOMA IR usually in women with body mass index (BMI) ≥ 25 kg/m^2^ and/or PCOS.

### 2.3. Laboratory Methods

Hormonal profile was measured by chemiluminescence using IMMULITE 2000 Immunoassay System (Siemen’s Healthcare Diagnostics Inc., Deerfield, IL, USA). Sensitivity (S) and the coefficient variation (CV) of each assay were for TSH, S: 0.004 μIU/mL, and CV: 5.1–12.5%; total-T3, S: 19 ng/mL and CV: 5.3–15.0%; fT4, S: 0.11 ng/dL and CV: 3.6–10.2%; insulin, S: 2 μIU/mL and CV: 4.1–7.3%; PRL S: 0.5 ng/mL and CV: 4.0–5.3%; P, S: 0.1 ng/mL and CV: 9.5–21.7%).

The IMMULITE 2000 also was used to determine TPO-Abs; the minimum detectable value for this technique is 10 IU/mL and the highest 1000 IU/mL; a value is considered positive if greater than 35 IU/mL. For TG-Abs the calibration range with this method is as high as 3.000 IU/mL, and the detection limit 20 IU/mL; TG-Abs values greater than 40 IU/mL were considered positive. Fasting glucose was measured using the Vitros DT60 II chemistry system (Ortho-Clinical Diagnostics, Tilburg, The Netherlands) with an S of 1.11 nmol/L and CV of 1.4-1.8% by the glucose oxidase method. HOMA IR was calculated using the formula (fasting insulin IU/mL × fasting glucose mg/dL)/405; the criteria for insulin resistance was a HOMA index > 2.5.

### 2.4. Study Variables and Endpoints

SCH was defined as TSH above the reference range and normal serum concentrations of FT4, with and without symptoms of hypothyroidism. The normal reference ranges for fT4 and tT3 were between 0.8–1.9 ng/dL and 70–170 ng/dL, respectively. As the primary endpoint, the prevalence of SCH was calculated using 2 different cut-off points for TSH: ≥2.5 mIU/L and ≥4.1 mIU/L.

As secondary endpoint, participants were divided into three study groups: Group 1, comprising women with TSH levels ranging between 0.3 and 2.49 mIU/L (control group); Group 2, women with TSH between ≥2.5 and 4.09 mIU/L; and Group 3, women with TSH ≥4.1 mIU/L. The clinical characteristics of the three groups were compared for age (years), height (m), weight (kg), body mass index (BMI kg/m^2^), years of infertility, type of infertility (primary or secondary), diagnosis of PCOS according to the Rotterdam criteria [24], oligo-anovulation (menstrual cycles longer than 35 days) and biochemical characteristics, i.e., TSH, fT4, TT3, TPO-Abs (+) and TG-Abs (+). Prolactin, progesterone, glucose, insulin and HOMA-IR.

### 2.5. Sample Size

To find an SCH prevalence of 15% using the cut-off for TSH ≥ 4.1 mIU/L, with a 95% confidence level and a 2% error, the sample size required was 1225 women.

### 2.6. Statistical Analysis

The statistical analysis was performed using the Statistical Package for Social Science Software (SPSS version 23, IBM Corporation, Chicago, IL, USA). Continuous variables were expressed as mean ± standard deviation or median and interquartile range, according to data distribution; categorical variables were reported as frequency, and proportions to compare continuous variables between groups were performed using one-way ANOVA test, with the Bonferroni correction or the Kruskal–Wallis test according to the distribution of each variable; the chi-square test or Fisher’s exact test were used to evaluate differences in proportions. Prevalence was calculated with a confidence interval (CI) 95%. Statistical significance was set at *p* ≤ 0.05.

## 3. Results

### 3.1. Participants

**Figure 1:**
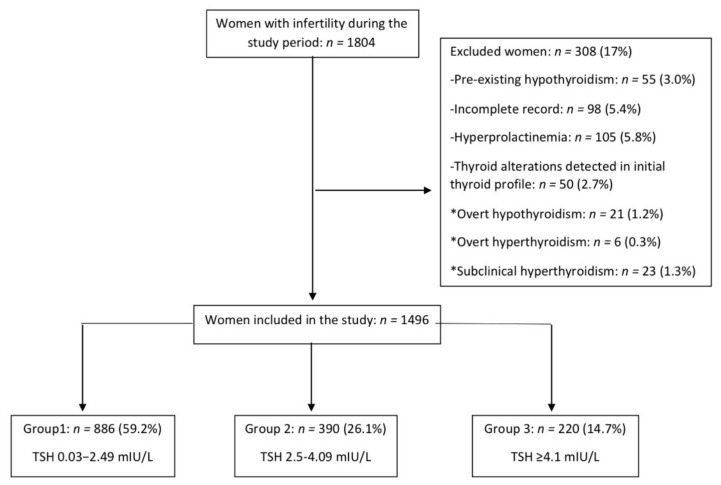
Flow-chart of participants and prevalence of SCH and other thyroid alterations among infertile Mexican women.

Over the study period, 1804 women were referred to our Infertility Clinic. At the initial analysis of the internal database, as many as 308 women were excluded, 98 because of incomplete medical records, 105 because they did not have study protocol of hyperprolactinemia, 55 due to pre-existing diagnosis of hypothyroidism and 50 because thyroid alterations were detected in their initial thyroid profile. A total of 1496 women were included; of these, 886 were included in Group 1 (TSH 0.3–2.49 mIU/L), 390 in Group 2 (TSH 2.5–4.09 mIU/L) and 220 in Group 3 (TSH ≥ 4.1 mIU/L); Figure 1.

### 3.2. SCH Prevalence and Thyroid Profile

The prevalence of SCH using a cut-off for TSH ≥ 2.5 mIU/L was significantly higher than with a cut-off for TSH ≥ 4.1 mIU/L: 40.7% (CI 95% 38.3–43.3%) vs. 14.7% (CI 95% 12.7–16.5%), (*p*= 0.0001); relative risk: 2.77; (CI 95% 2.4–3.2). Mean TSH values were increased significantly among Groups 1, 2 and 3, respectively. Significant differences were found between groups for serum levels of total-T3 and fT4 (Table 1). T3T concentrations increased progressively in Groups 1,2, and 3, respectively, while fT4 decreased.

Thyroid autoantibodies were analyzed in 115 patients in Group 1, 252 in Group 2 and 170 in Group 3. We found no significant differences in terms of thyroid autoimmunity (TPO-Abs and TG-Abs) prevalence between Group 1 versus Group 2 (*p* > 0.05). As we expected, we found a significantly higher prevalence of thyroid autoimmunity (anti-TPO) in Group 3 versus Group 1. (*p* = 0.03); Table 1.

### 3.3. Clinical Features

There were no significant differences among the three groups in terms of age, height, duration and type of infertility (primary or secondary), and prevalence of oligo-anovulation or polycystic ovary syndrome. However, we found a significant difference in terms of weight between Group 1 and Group 2 and even more significant between Group 1 and Group 3. The prevalence of overweight women was significantly higher in Group 2 than in Groups 1 and 3. The prevalence of obesity was higher in Group 3 than Groups 1 and 2 (*p* = 0.0001); Table 2.

### 3.4. Other Biochemical Features

Insulin serum values and HOMA-IR were significantly higher in Groups 2 and 3 versus Group 1, as was the rate of insulin resistance (*p* < 0.05). No other significant differences were observed among groups (see for details Table 3).

## 4. Discussion

In the present study, we show that changing the TSH cut-off from ≥4.1 mIU/L to ≥2.5 mIU/L significantly increases the prevalence of SCH in infertile Mexican women. Using the 2.5 mUI/mL cut-off limit, the number of women classified as SCH increased nearly three-fold compared with the most accepted 4.1 mIU/L cut-off limit [8,12,14,15]. These differences have a relevant psychosocial and economic impact and might be related to selection criteria, autoimmune thyroid disease, TSH isoforms, ethnic origin, iodine intake and analytical methodology.

According to published observational studies in unselected populations of infertile women, the prevalence of SCH is highly variable, ranging from 0.7 to 43% [6,13,14,15,16,25,26]. The effects of this condition on reproductive dynamics are uncertain, and there is no consensus over treatment [1,2,3,4,5,6,7,8,9,10]. Most studies in women with infertility set a limit TSH >4.1 mIU/L as a diagnostic standard for SCH [8,12,25,26]. According to this cut-off, 3−34% of women seeking pregnancy carry this condition [12,14,16,25,26,27]

Our findings are different from other observational studies that adopt a TSH > 4.1 mIU/L. In our center, the prevalence was 14.7%, similar to the study by Abolovich et al. in a sample of infertile women from a public hospital in Argentina (*n* = 244) [25] and higher than the prevalence reported by Shalev et al. in 444 infertile Israeli women (0.7%) with TSH ˃4.5 mIU/L [15]. Likewise, Grassi et al. [26], found a prevalence of 4.6% in 149 Italian infertile women with a cut-off of TSH ≥ 4.5 mIU/L, and Poppe et al. reported a prevalence of 0.9% in 438 Belgian women using a cut-off of TSH ≥ 4.2 mIU/L [8]. On the other hand, Raber et al., reported in Austrian infertile women a 26.85% prevalence of SCH using an upper limit of reference TSH ≥ 4.0 mIU/L, which is over two-fold higher than our prevalence with the same cut-off [14].

We failed to find national reports comparing the prevalence of SCH among infertile women in other centers in Mexico. Mendez-Villa et al. reported an SCH prevalence (using TSH cut-off > 4.5 mIU/L) of 2.9% among 101 healthy Mexican women of childbearing age (mean age: 21.7 ± 3.5 years) [17]. Data about normal TSH reference values for the Mexican population are limited; indeed, Flores-Rebollar et al. reported that normal TSH reference in a group of 127 iodine-sufficient Mexican women of childbearing age without thyroiditis and normal thyroid ultrasound, who met the NCAB criteria, corresponds to 0.69–5.34 mIU/L (percentile 2.5 to 97.5th); however, these findings are flawed due to the fact that the study was underpowered [18].

Otherwise, TSH levels are directly related to the degree of iodine nutrition of the population [28]. There is a close relationship between iodine intake and the risk of developing thyroid disease, which tends to appear in instances of both excessive and deficient iodine intake [29]. Although the present study did not document the iodine intake in the participants, a recent study reported adequate iodine intake in 94% of healthy adults in Mexico City, which could be similar to our study population [30]. The prevalence of thyroid dysfunction reported among women in this study was 12.5%; however, it failed to demonstrate the association of thyroid dysfunction with iodine intake status in the Mexican population [30].

Analyzing current evidence shows that the pharmacological intervention in infertile women with mild isolated SCH (TSH 2.5−4 mIU/L) is controversial because SCH autoantibody–negative infertile women not undergoing ART were not included in controlled trials; therefore, there is no evidence to support the use of LT4 therapy in these women [2,3,4,5,6,7]. Some international associations support treatment in infertile women with TSH between 2.5–4.5 mIU/L and positive thyroid antibodies to prevent the progression to more significant hypothyroidism once pregnancy is achieved, given the minimal risk associated with this treatment [1,3]. Conversely, based on the results of observational studies, other international associations do not recommend treatment in this population [9,10,20].

Reh et al. demonstrated, in 1055 infertile U.S. women candidates for ART, that using a cut -off of TSH ≥ 2.5 mIU/L increased by four-fold the prevalence of SHC compared to a cut-off of TSH ≥ 4.5 mIU/L, without significant difference in clinical pregnancy, abortion and live birth rates [16]. Likewise, in a randomized trial whose primary aim was to evaluate the effect of LT4 in a sample of infertile women treated with IVF/ICSI (*n* = 32) and isolated concentrations of TSH > 4.5 mIU/L, Kim CH et al., reported better embryo quality, higher implantation rate and live births in the group treated compared with a control group; however, the sample size of the study was small [21]. Finally, a meta-analysis including data from 220 women indicated that levothyroxine treatment with TSH ≥ 4 mIU/L during assisted fertilization techniques was associated with statistically significantly lower miscarriage rates (RR = 0.45; 95% CI: 0.24–0.82) but failed to find an association between treatment and clinical pregnancy rates (RR 1.75; 95% CI 0.90–3.38) [22].

The potential pitfalls of universal screening followed by treatment include increased health costs and concerns about safety [4,5]. Patients started on LT4 therapy need to be monitored closely to ensure that they are euthyroid and prevent overtreatment. This can lead to multiple appointments at primary or secondary care settings, create anxiety, and delay attempts to conceive or seek fertility treatment [6,7].

As to metabolic variables, our study showed a significant association between SCH and insulin resistance, although we attributed this finding to a higher prevalence of obesity among SCH women; conversely, Benetti-Pinto et al. failed to find a higher incidence of insulin resistance in a sample of Brazilian women presenting with PCOS and SCH, but the strength of their findings was limited by the sample size [31].

The prevalence of SCH has been reported to be higher in general in obese patients, which is consistent with our results [32]. A positive correlation between serum levels of TSH and BMI has been described in cross-sectional population studies [33]; this has been attributed to the hypothalamus’s adaptive response–pituitary–thyroid axis to weight gain [34]. Weight gain is also associated with higher T3 production, with no change in serum levels of T4 or fT4, and obese patients show higher TSH and FT3 or T3/FT4 ratios, which suggests an increased conversion of T4 to T3 due to increased deiodinase activity as a compensatory mechanism to improve energy expenditure [33,35]. As expected, obesity was more prevalent in women with higher TSH concentrations (Group 3), and these women also had higher T3 concentrations. However, as TSH concentrations increased between the groups, we also observed a modest decrease in fT4 concentrations, which cannot be attributed to obesity. Group 3 also had a higher prevalence of autoimmune thyroid disease, which could explain the lower fT4 concentrations.

It has recently been questioned whether it is appropriate to diagnose hypothyroidism based only on abnormal TSH levels; one recent meta-analysis [36] shows that thyroid hormone levels (T3 and FT4) correlate better with clinical parameters than TSH levels. It is uncertain if the differences in fT4 concentrations observed between the three groups could have a clinical meaning in obstetrics or fertility outcomes. Future studies could explore if FT4 concentrations in women with SCH can predict which women could benefit from treatment better than TSH can.

The limitations of our study are that the data collection was retrospective, that it did not have a second quantification of TSH, and that thyroid autoimmunity and insulin resistance were not measured on the entire population and there is no national reference for thyroid test in Mexican women. Although insulin resistance was measured in 70% of women in each study group, the sample had adequate power for valid comparison among groups. However, the thyroid antibodies were only measured in 13% of patients in Group 1, compared to 64% and 77% in Groups 2 and 3, respectively. This could limit the comparison of thyroid autoimmunity among groups. The main strengths of our study are the sample size and the fact that, to the best of our knowledge, it is the first study to report the prevalence of SCH among infertile Mexican women.

Nevertheless, randomized clinical trials and cost–benefit studies are required to evaluate the effect of treatment in infertile women with SCH using different cut-off levels for TSH.

## 5. Conclusions

The prevalence of SCH among infertile Mexican women using a TSH cut-off ≥ 2.5 mIU/L was almost three times higher than with a TSH cut-off ≥ 4.1 mIU/L. Infertile women with TSH ≥ 4.1 mIU/L were more often obese and insulin-resistant than women with TSH ≤ 2.5 mIU/L.

## Figures and Tables

**Table 1:** Thyroid profile and thyroid antibodies of infertile Mexican women with and without subclinical hypothyroidism using two cut-offs for TSH.

Thyroid Determination	Group 1 (TSH 0.3–2.49 mIU/L) *n* = 886	Group 2 (TSH 2.5–4.09 mIU/L) *n*= 390	*p* Group 1 vs. 2	Group 3 (TSH ≥ 4.1 mIU/L) *n* = 220	*p* Group 1 vs. 3
TSH (mUI/L)	1.55 ± 0.5	3.1 ± 0.4	0.0001	5.6 ± 1.3 *	0.0001
T3T (ng/dL)	122 ± 28	128 ± 28	0.001	130 ± 28	0.0001
fT4 (ng/dL)	1.29 ± 0.29	1.23 ± 0.24	0.0001	1.16± 0.21 *	0.0001
Thyroid antibody determination	*n* = 115 (13.0%)	*n* = 252 (64.6%)		*n* = 170 (77.3%)	
Positive TPO-Abs	*n* = 9 (7.8%)	*n* = 33 (13.1%)	0.19	*n* = 33 (19.4%)	0.01
Positive TG-Abs	*n* = 4 (3.5%)	*n* = 14 (5.5%)	0.55	*n* = 11 (6.5%)	0.4
Thyroid autoimmunity	*n* = 11 (9.6%)	*n* = 34 (13.5%)	0.37	*n* = 33 (19.4%)	0.03

* Group 2 vs. 3 *p* < 0.01. TSH = Thyroid-stimulating hormone. T3T = total triiodothyronine fT4 = free thyroxine TPO-Abs = anti-thyroperoxidase antibodies; TG-Abs = anti-thyroglobulin antibodies, BMI = body mass index. Value expressed as mean ± standard deviation, median and [interquartile range] or frequency and (percentage).

**Table 2:** Clinical characteristics of infertile Mexican women with and without subclinical hypothyroidism using two cut-offs for TSH.

Characteristic	Group 1 TSH 0.3−2.49 mIU/L *n* = 886	Group 2 (TSH 2.5−4.09 mIU/L) *n* = 390	*p* Group 1 vs. 2	Group 3 (TSH ≥ 4.1 mIU/L) *n* = 220	*p* Group 1 vs. 3
Age (years)	29.8 ± 3.9	30.1 ± 4.1	0.98	30.9 ± 4.8	0.27
Years of Infertility	5.1 ± 3	5.1 ± 3.1	0.98	5.1 ± 3	0.98
Weight (kg)	66.2 ±12	68.1 ± 12.9	0.03	70.3 ± 13.7	0.0001
Height (m)	1.55 ± 0.06	1.56 ± 0.06	0.41	1.56 ± 0.06	0.14
BMI (kg/m^2^)	27.3 ± 4.5	27.9 ± 4.8	0.11	28.7 ± 5.1	0.0001
Primary infertility	668 (75.4)	300 (76.9)	0.75	168 (76.4)	0.96
Secondary infertility	218 (24.6)	90 (23.1)	0.75	52(23.6)	0.96
Normal weight (BMI 18.5−24.99 Kg/m^2^)	299 (33.7)	103 (26.4)	0.01	58 (26.4)	0.04
Overweight (BMI 25−29.99 Kg/m^2^)	354 (40)	184 (47.2)	0.01	77 (35) *	0.20
Obesity (BMI ≥30 Kg/m^2^)	233 (26.3)	103 (26.4)	0.97	85 (38.6) *	0.0001
Oligo-anovulation	438 (49.4)	202 (51.8)	0.47	111 (50.5)	0.84
Polycystic ovarian syndrome	290 (32.7)	141 (36.2)	0.26	80 (36.4)	0.34

* Group 2 vs. 3 *p* < 0.01. TSH = thyroid-stimulating hormone. BMI = body mass index. Value expressed as mean ± standard deviation, median and [interquartile range] or frequency and (percentage).

**Table 3:** Biochemical characteristics of infertile Mexican women with and without subclinical hypothyroidism using two cut-offs for TSH.

Characteristics	Group 1 TSH 0.3−2.49 mIU/L *n* = 886	Group 2 (TSH 2.5−4.09 mIU/L) *n* = 390	*p* Group 1 vs. 2	Group 3 (TSH ≥ 4.1 mIU/L) *n* = 220	*p* Group 1 vs. 3
Prolactin (ng/mL)	12.1 (9.2–16)	12.4 (9.2–17)	0.55	12.7 (9.3–17.7)	0.24
Progesterone (ng/mL)	3 (0.65–11.1)	1.8 (0.52–10.6)	0.16	1.37 (0.56–9.8)	0.08
Women with glucose and insulin	*n*= 616 (69.5)	*n* = 271 (69.5)		*n*= 155 (70.4)	
Glucose (mg/dL)	93.1 ± 17	93.8 ± 18	0.98	93.6 ± 11	0.96
Insulin (µU/mL)	8.9 (5.8–12.6)	10.8 (7.7–16.7)	0.004	12 (7–18.9)	0.0001
HOMA-IR	2.1 (1.2–3.4)	2.4 (1.4–4.0)	0.008	2.7 (1.7–4.3)	0.0001
Insulin resistance	246 (40)	125 (46.1)	0.09	84 (54.2)	0.001

Group 2 vs. 3 no significant differences. TSH = thyroid stimulating hormone. Value expressed as mean ± standard deviation, median and [interquartile range] or frequency and (percentage).

## Data Availability

Please contact the corresponding author for data requests.
